# Identifying patient-specific behaviors to understand illness trajectories and predict relapses in bipolar disorder using passive sensing and deep anomaly detection: protocol for a contactless cohort study

**DOI:** 10.1186/s12888-022-03923-1

**Published:** 2022-04-22

**Authors:** Abigail Ortiz, Arend Hintze, Rachael Burnett, Christina Gonzalez-Torres, Samantha Unger, Dandan Yang, Jingshan Miao, Martin Alda, Benoit H. Mulsant

**Affiliations:** 1 Department of Psychiatry, University of Toronto, Toronto, Ontario Canada; 2 Centre for Addiction and Mental Health (CAMH), 100 Stokes St., Rm 4229, Toronto, ON Canada; 3 Department of Computer Science, Dalarna University, Dalarna, Sweden; 4 Department of Pharmacology, University of Toronto, Toronto, Ontario Canada; 5 Department of Psychiatry, Dalhousie University, Halifax, Nova Scotia Canada; 6 National Institute of Mental Health, Klecany, Czech Republic

**Keywords:** Bipolar disorder, Episode prediction, Wearable device, Machine learning

## Abstract

**Background:**

Predictive models for mental disorders or behaviors (e.g., suicide) have been successfully developed at the level of populations, yet current demographic and clinical variables are neither sensitive nor specific enough for making individual clinical predictions. Forecasting episodes of illness is particularly relevant in bipolar disorder (BD), a mood disorder with high recurrence, disability, and suicide rates. Thus, to understand the dynamic changes involved in episode generation in BD, we propose to extract and interpret individual illness trajectories and patterns suggestive of relapse using passive sensing, nonlinear techniques, and deep anomaly detection. Here we describe the study we have designed to test this hypothesis and the rationale for its design.

**Method:**

This is a protocol for a contactless cohort study in 200 adult BD patients. Participants will be followed for up to 2 years during which they will be monitored continuously using passive sensing, a wearable that collects multimodal physiological (heart rate variability) and objective (sleep, activity) data. Participants will complete (i) a comprehensive baseline assessment; (ii) weekly assessments; (iii) daily assessments using electronic rating scales. Data will be analyzed using nonlinear techniques and deep anomaly detection to forecast episodes of illness.

**Discussion:**

This proposed contactless, large cohort study aims to obtain and combine high-dimensional, multimodal physiological, objective, and subjective data. Our work, by conceptualizing mood as a dynamic property of biological systems, will demonstrate the feasibility of incorporating individual variability in a model informing clinical trajectories and predicting relapse in BD.

## Background

Bipolar disorder (BD) is a mood disorder with high recurrence and disability rates [1]. It is estimated that the suicide risk in patients with BD is 20 times higher than in the general population [2]. A key feature of BD is its varying severity over time, in some patients manifested as distinct mood episodes, in others as fluctuating intensity of mood symptoms. As a result, interventions for long-term treatment and relapse prevention are crucial for the clinical management of patients with BD [3]. However, these strategies have not decreased the incidence of relapse or adverse outcomes [4]. One of the reasons relapse prevention may not be successful is because traditional clinical monitoring is not frequent enough to manage the capricious nature of the illness [5].

Similarly, predictive models for mental disorders or behaviors (e.g., suicide) have been successfully developed at the level of populations, yet current demographic and clinical variables are neither sensitive nor specific enough for making individual clinical predictions. For instance, a recent meta-analysis concluded that predictive ability for dying from suicide has not improved over 50 years of research [6]. New analytical approaches based on nonlinear mathematical methods (i.e., time-series analyses and entropy calculations) and machine learning (anomaly detection), coupled with passive sensing (i.e., wearables) may allow us to address this problem, which has been intractable so far.

The wide availability of low-cost wearables enables us to measure how the environment and our experiences impact our physiology. Recent studies in electronic (e-)monitoring in BD have typically focused on group differences rather than improving outcomes [7]. Also, these studies have faced two major challenges: (i) a high proportion of missing data; (ii) and inadequate statistical tools to process large amounts of data. Moreover, participants identify forgetfulness as the top barrier for engagement with e-monitoring [8]. Thus, passive sensing, which requires minimal effort from participants, may lead to more effective data collection.

While most studies concur on the difficulty of finding an overarching prediction algorithm that is applicable to a wide variety of patients, individual variability is a concept that has not been taken into account, even though it is the most important factor in individualized risk estimation [9, 10]. We hypothesize that we will not be able to make actionable individual predictions for patients with mental disorders without characterizing individual variability [10]. Thus, to understand the dynamic changes involved in episode generation in BD, we propose to integrate physiological, objective, and subjective clinical variables relevant for mood regulation using nonlinear techniques. We hypothesize that integrating these variables will allow us to assess the impact of individual variability on illness trajectories and predict the onset of episodes in BD. Here we describe the study we have designed to test this hypothesis and the rationale for its design. We also illustrate the analytical process involved in deep anomaly detection, which is the technique we will use to forecast episodes of illness.

## Methods

### Study overview

The study will recruit 200 participants diagnosed with BD I or BD II. After completing a comprehensive baseline assessment, they will be followed for up to 2 years during which they will be monitored continuously with a wearable Oura ring (Oura Health Oy, Generation 2, Oulu, Finland) and will complete questionnaires regularly. The study will be contactless, i.e., all the research procedures will be conducted by phone or virtually. Oura Health Oy did not sponsor the study nor has access to collected data stemming from this study.

### Settings

Participants will be recruited at two academic centers in Canada: the Adult Psychiatry Division, Centre for Addiction and Mental Health (CAMH), Toronto, Ontario; and the Mood Disorders Program, Queen Elizabeth II Health Sciences Centre, Halifax, Nova Scotia. The study has been approved by the local REBs with extensive feedback from the Information Technology (IT) and Privacy leaders.

### Recruitment and consent

We will include adult men or women of any race and ethnicity, aged 18 and older (with no upper age limit), with a primary diagnosis of BD I or II, according to DSM-5 criteria [11] based on the SCID-5 [12], in any phase of the illness (i.e., euthymic, depressive, (hypo)manic, or mixed). Treating physicians at the two academic centers will refer their potentially eligible patients who express an interest in participating in the study. All participants will provide written informed consent using a form approved by the local REB and will be advised of their right to withdraw from the study.

### Baseline assessment and clinical follow-up

After providing informed consent, participants will complete a comprehensive baseline assessment as summarized in Table 1. This baseline assessment includes six domains: socio-demographic, diagnosis, clinical course, cardiovascular screening, chronotype determination, and pharmacotherapy. Collecting this information will allow us to characterize the sample and assess variables that will be included in the predictive model, including Illness Burden Index [13]. Participants will receive standard (“as usual”) medical treatment.

**Table 1:** Baseline variables collected to characterize sample

Domain	Instrument	Main variables	Use of data
Socio-Demographics	Socio-demographic questionnaire	Self-reported gender, race/ethnicity, age, occupation, number of years of education, marital and work status	Characterization of the sample and variables included in predictive model
Diagnosis	SCID-5, MADRS, YMRS	Diagnosis of BD I or II; list of depressive or manic symptoms	Confirmation of diagnosis and characterization of polarity upon entry to the study
Clinical Course	Clinical questionnaire	Age at onset, number and type of previous episodes, history of suicide attempts, history of psychotic symptoms during episodes, co-morbid disorders, number of lifetime admissions, family history of any psychiatric disorder in first-and second-degree relatives, pattern of mood reactivity (history of antidepressant-induced (hypo)manias, rapid cycling)	Characterization of course of illness (episodic with or without residual symptoms, chronic fluctuating and chronic) and calculation of illness burden index, and variables included in predictive model
Cardiovascular screening	Clinical questionnaire, DASI	BMI, smoking history, presence of kidney disease, family history of angina or myocardial infarction in a first-degree relative before the age of 60	Characterization of the sample
Chronotype	MEQ-19	Morning, intermediate, or evening chronotype	Characterization of the sample and variables included in predictive model
Pharmacotherapy	Clinical questionnaire	Name, dosage, and date medication(s) started	Characterization of the sample and variables included in predictive model

*BD* Bipolar disorder, *BMI* Body mass index, *DASI* Duke Activity Scale Index, *IBI* Illness Burden Index, *MADRS* Montgomery-Asberg Depression Rating Scale, *MEQ-19* Morningness-Eveningness Questionnaire, 19 items, *SCID*: 5 Structured Clinical Interview for DSM-5, *YMRS* Young Mania Rating Scale

A trained rater will administer the Young Mania Rating Scale (YMRS) [14] and the Montgomery-Asberg Depression Rating Scale (MADRS) [15] to determine polarity at study entrance. Euthymia will be operationalized as a score ≤ 10 on both scales. MADRS scores will be used to define the presence and severity of depressive episodes as per established guidelines [16] (mildly ill: 11–18; moderately ill: 19–23; moderate to severely ill: 24–36; severely ill: 37–39; or extremely ill: ≥ 40). Similarly, YMRS scores will be used to define episodes of hypomania, mania, or mania with psychosis (hypomania: 11–19; mania; ≥ 20, with or without psychotic symptoms). Both scales will be used in all participants because mixed episodes may be difficult to detect in the early stages. Cardiovascular (functional) capacity will be assessed with the Duke Activity Status Index Scale (DASI) [17], a 12-item questionnaire that assesses a person’s ability to perform a set of activities (personal care, ambulation, household tasks, sexual activity, recreational activity). The DASI scores integrates weighted answers based on the known metabolic cost for each activity in Metabolic-Equivalent Task (MET) units [18]; it ranges from 0 to 58.2, with higher values indicative of higher (better) functional capacity. Chronotype will be assessed with the 19-item Morningness-Eveningness Questionnaire (MEQ) [19], the responses of which are combined into a composite score that indicates participant’s chronotype (morning, intermediate or evening). Finally, we will classify medications (and changes in medications), as we have done in previous studies, based on class, dosage, and duration of treatment [13].

### Passive sensing

Participants will use the Oura ring (ouraring.com), a titanium-made ring weighing 4–6 g that collects data automatically and transfers them via Bluetooth to a smartphone on which a specialized app has been loaded; in turn, that app transfers the data to a centralized database maintained by the manufacturer and accessible to the research team. The ring assesses activity and sleep with a 3-D accelerometer and gyroscope, and heart rate (HR) and heart rate variability (HRV) with an infrared optical pulse measurement. Total sleep time and sleep latency determined by the ring are correlated 0.93 with polysomnography [20].

Participants will be sent a sizing kit to determine their ring size; they will be encouraged to try several sizes for a day or two, including during their sleep, to ensure a comfortable fit. Once participants confirm their size, we will mail them the actual wearable and they will be asked to wear it continuously for the duration of the study. As summarized in Table 2, we will use this wearable to objectively and continuously track some measures related to mood regulation: HR, HRV; parameters related to sleep (e.g., total sleep and REM-sleep duration), activity (e.g., number of steps during the day and of active and inactive minutes), and energy (e.g., energy consumption in MET during the day).

**Table 2:** Physiological, objective, and subjective variables collected during the study

	Continuous e-monitoring (Oura Ring)	Self-reports (DASI, MEQ, ASRS, PHQ-9, Visual analog scale)	Clinician ratings (MADRS, YMRS)
**Physiological**	**HR and HRV**: average HR for each 5 mins of sleep period; lowest HR registered during sleep; average HR during the sleep period; HRV using RMSSD during the night and for each 5 mins of sleep period.		
**Objective**	**Sleep**: local time when sleep starts and ends; onset latency; total duration of sleep; total duration of awakenings during sleep period; sleep efficiency; total amount of light, deep- and REM-sleep; restlessness (percentage of sleep time when movement was detected). **Activity**: local time when local activity starts and ends; number of minutes during the day with low, medium, and high activity; number of inactive minutes during the day; number of minutes during the day when the user was resting (lying down or napping); number of minutes during the day when the user was not wearing the ring; total number of steps during the day. **Energy**: energy consumption (MET) during low, medium, and high activity periods and during the day.		**Baseline and end of study**: MADRS: observed sadness. YMRS: observed motor activity, irritability, rate and amount of speech, disordered language or thought, thought content, disruptive or aggressive behavior, appearance, insight. **Baseline and throughout the study:** Pharmacotherapy questionnaire: name, dose, and date medication started or changed; medication compliance
**Subjective**		**Baseline:** DASI: 12-item questionnaire to assess ability to perform a set of activities (personal care, ambulation, household tasks, sexual activity, recreational activity) to gauge functional cardiovascular capacity. MEQ: 19 multiple-choice questions to determine chronotype. **Baseline and weekly:** ASRS: 6-item to assess mood, sleep, activity, grandiosity, and talkativeness. PHQ-9: 9-item to assess mood, pleasure (anhedonia); sleep, energy, appetite, guilt, concentration, psychomotor retardation or agitation, thoughts of death or suicide and their impact on functioning. **Daily:** Visual analog scale: self-rated fluctuations in mood, anxiety, and energy levels for each day	**Baseline and end of study**: MADRS: reported sadness, inner tension, sleep, appetite, concentration, lassitude, inability to feel, pessimism, suicidal thoughts. YMRS: mood, energy, sexual interest, sleep, irritability

*ASRS* Altman Self-Rating Mania Scale, *DASI* Duke Activity Status Index Scale, *HR* heart rate, *HRV* heart rate variability, *MADRS* Montgomery-Asberg Depression Rating Scale, *MET* Metabolic Equivalent of a Task, *MEQ* Morningness-Eveningness Questionnaire, *PHQ-9* Patient Health Questionnaire, 9 items, *REM* Rapid Eye Movement, *RMSSD* Root Mean Square of Successive Differences, *YMRS* Young Mania Rating Scale

### Follow-up ratings

As shown in Table 2, participants will rate their mood, anxiety, and energy level daily using electronic visual analog scales (e-VAS) accessed via an e-mailed link. The scale ranges from ‘1’ to ‘9’, with ‘5’ being “your usual”, 1 “your lowest”, and 9 “your highest” and participants describe with a swipe of their finger on the e-VAS their mood, anxiety, or energy level throughout the day, according to the Day Reconstruction Method [21]. These e-VAS, which we used in our previous studies with a paper-version, use a densely-sampled scale (change interval is 0.1), which allows us to generate continuous, fine-grain data. We will use interpolation methods when data are missing or incomplete for 1 or 2 days in a row. When data are missing for 3 days, we will contact participants by text or email and remind them about the importance of filling out the scale on a daily basis. In addition, every week, participants will complete the Patient Health Questionnaire (PHQ-9) [22] and the Altman Self-Rating Mania Scale (ASRS) [23]. Participants will get a secure email with a link to both scales and complete self-ratings, which will be uploaded into REDCap.

### Data collection

All study data will be managed using REDCap electronic data capture tools hosted at CAMH. REDCap (Research Electronic Data Capture) is a secure, web-based software platform designed to support data capture for research studies, providing: an intuitive interface for validated data capture; audit trails for tracking data manipulation and export procedures; automated export procedures for seamless data downloads to common statistical packages; and procedures for data integration and interoperability with external sources [24].

### Statistical analyses

First, a descriptive analysis will characterize the participants at baseline and summarize missing data. Then we will compare the baseline characteristics based on the outcome groups: “becoming ill” or “not becoming ill”. We will also use graphs to present the data and individual trajectories. We will evaluate the association among time-varying variables (i.e., sleep, mood, activity). Traditional statistical techniques will be used to model the data for comparative purposes. Cox Regression models adjusted for baseline and time-varying variables will be used to model the time until events (e.g., relapse). We will use time-dependent covariates and the occurrence of the event as censoring. We will also use dynamic time-series to fit independent models for each participant. Finally, because negative correlations have been found between age or socioeconomic status, and use of health apps [25], we will incorporate self-reported gender, age, and other relevant factors (e.g., years of education) in all our analytical models and assess whether any of these influence our results. Statistical analyses will be done using MATLAB®.

### Sample size calculation

Previous studies report that up to 44–48% of BD patients relapse in the first 2 years of follow-up, and that depressive episodes are more common than hypomanic or manic ones [26]. With 200 participants with BD receiving standard treatment and followed for 12 months, we expect at least 60 participants to experience the onset of a new depressive episode and 10 to experience the onset of a new hypomanic/manic episode throughout the study duration (i.e., 35% of the sample). In our previous study, entropy calculations for the mood series showed a normal distribution with mean (SD) of 1.04 (0.6) in 30 BD patients and 1.47 (0.3) in 30 healthy comparators. Thus, if we replicate these findings in our 200 participants (H1a), we will have a power of 0.90 to differentiate highly correlated series (i.e., equivalent to low entropy levels). While this project has not been powered for H2, if the true difference in entropy levels between euthymia and illness is 0.22, we will have a power of 0.80 to reject the null hypothesis for H2 (with a Type I error probability of 0.05).

### Nonlinear analyses: time-series analyses and entropy calculations

For all the time-series generated (e.g., sleep), we will compute auto-correlation for each series (i.e., how one point in the series correlates to the next point in the same series); and cross-correlation between all series (i.e., how one point in one series correlates with a corresponding point in a different series). Furthermore, we will analyze whether these variables: (i) differentially contribute to the model to identify clinical trajectories; (ii) differentially predict events (e.g., relapse). Entropy calculations for each variable will be continuously recomputed from each of the time-series. In turn, as described below, time series-data from each of the above-mentioned variables will be fed into our machine learning technique one step at a time.

### Outlier and anomaly detection

The use of digital analytics for precision health is now possible given advances in machine learning models that can extract complex patterns from multiple sources of high-dimensional data over time. However, understanding how some machine-learning techniques work remains a challenging task, as it has an inherent “black box” character. Thus, we propose to use deep learning anomaly detection, a machine learning technique that doesn’t have this limitation. The principle behind outlier detection is simple and well established [27]. While this approach works robustly for single variables, once multidimensional data is considered, the deviation from the mean is not as easily computed. Scaling, distribution, and, most importantly for this study, the temporal sequence of values matters greatly. Thus, by using deep anomaly detection, instead of computing a mean and the deviation from the mean, a deep neural network can be trained on the data. The model makes predictions and the deviation of the model’s predictions from the observed participant identifies anomalous behavior. To date, deep anomaly detection has been mostly applied in risk management, security and financial surveillance [28].

We will use two different types of anomaly detection techniques: autoencoders and recurrent neural networks (RNN) for time-series prediction. We will use both methods because they differ in what they predict: an autoencoder takes a time-series (or pattern) as an input and tries to generate the same series or pattern as an output. While this sounds trivial, if the autoencoder is large enough, it becomes challenging, because the autoencoder has an information bottleneck. This bottleneck consists of very few nodes, and requires the data to be compressed, losing information, and only allowing major trends to pass through. Conversely, when using RNN or their more advanced variants, such as Long Short-Term Memory (LSTM) [29], the data is fed into the model one time step after the other, while the model continuously predicts the following time point.

**Fig. 1:**
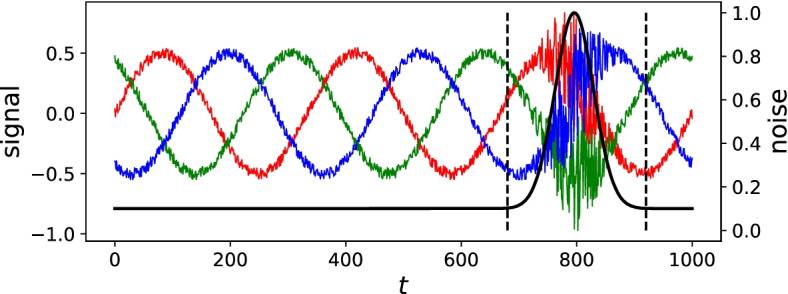
Using deep learning models with data containing different signals. An example of using deep learning models with data containing three different signals. This data, while following a sinus rhythm with low noise (red, green, and blue lines), experiences high noise at the end of the series (solid black line for noise and dashed lines identifying outlier period)

Figure 1 illustrates this process with a simulated dataset including three different signals (e.g., heart rate, activity, and sleep). A RNN was trained on this dataset (including the outlier period) to predict the next data points based on the last 20 time points. After successful training, the difference between the model’s prediction and the actual data was computed for all time points. The distribution of that deviation allows us to determine a threshold, i.e., 95% confidence interval. All data points for which the prediction is aberrant from the observation successfully identified the period in which the anomaly is expected (figure not shown). The RNN was trained on the data containing the outlier period, as this would be the case with participants’ data, which will also contain outlying periods.

## Discussion

This proposed contactless, large cohort study aims to obtain and combine high-dimensional, multimodal physiological (HRV), objective (sleep, activity), and subjective data using passive sensing and e-rating scales (self-ratings and clinician-rating scales). The overall objective of the study is to extract and interpret individual illness trajectories and patterns suggestive of relapse using deep anomaly detection. Our Methods are predicated on the following: (i) models do not generalize from one participant to another; (ii) model hyper-parameters that optimally predict one participant’s trajectory (including outliers) may be different from participant to participant; (iii) a single model trained on all participants’ data cannot be used to identify outliers. Moreover, unlike many models generated by other machine learning techniques, the resulting model would be understandable and interpretable (i.e., it would not be a “black-box”). The integration of densely-sampled (i.e., “deep” data) objective and autonomic data with each participant’s unique contextual information will further enhance the performance of the model.

Potential impediments to the success of this study include (i) some participants will not be willing or able to use the wearable; (ii) relapse rates may be too low over the length of the study; (iii) participant dropout. To minimize the impact of these potential impediments, we will monitor adherence and contact participants who have not uploaded data for 3 days. In addition, participants will have their usual, regular clinical follow-ups, which will give us an additional opportunity to encourage adherence with study procedures. However, participants are informed through the consent form that we are not able to monitor their illness in real time; and they are advised to obtain medical advice if they feel their mood is deteriorating. In these instances, we expect that most psychiatrists will promptly assess their patients to confirm whether they are indeed experiencing a relapse and will implement appropriate actions when warranted clinically.

The major strengths of our study include its foundation on a solid work of an interdisciplinary team on clinical trajectories in BD; nonlinear properties of mood regulation in healthy controls, BD patients, and their unaffected first-degree relatives [10, 30]; feasibility of using passive sensing in adults with BD during different types of episodes [13]; and a simulation using machine learning for outcome prediction [31]. By conceptualizing mood as a dynamic property of biological systems, we plan to demonstrate the feasibility of incorporating individual variability in a model informing clinical trajectories and predicting relapse in BD. Ultimately, if we succeed in predicting relapses, we should be able to prevent them.

## Data Availability

Not applicable.

## Abbreviations

ASRS: Altman Self-Rating Mania Scale
BD: Bipolar disorder
BMI: Body mass index
DASI: Duke Activity Status Index Scale
HR: Heart rate
HRV: Heart rate variability
IBI: Illness Burden Index
MADRS: Montgomery-Asberg Depression Rating Scale
MET: Metabolic Equivalent of a Task
MEQ: Morningness-Eveningness Questionnaire
PHQ-9: Patient Health Questionnaire, 9 items
REM: Rapid Eye Movement
RMSSD: Root Mean Square of Successive Differences
SCID: 5: Structured Clinical Interview for DSM-5
YMRS: Young Mania Rating Scale
